# Knowledge, attitudes, and practices regarding body weight management among patients with overweight or obesity: a cross-sectional study

**DOI:** 10.3389/fpubh.2025.1615478

**Published:** 2025-07-23

**Authors:** Wenjun Shi, Cuiliu Ma, Guishan Zhang, Zhijiao Xu, Xing Liu, Yang Li, Cuijing Zhang

**Affiliations:** ^1^Department of Clinical Nutrition, The First Hospital of Zhangjiakou, Zhangjiakou, China; ^2^Department of Endocrinology, The First Hospital of Zhangjiakou, Zhangjiakou, China; ^3^Department of Hepatological Surgery, The First Hospital of Zhangjiakou, Zhangjiakou, China

**Keywords:** weight management, obesity, overweight, knowledge, attitudes, practices, patient education

## Abstract

**Objectives:**

This study aimed to investigate the knowledge, attitudes, and practices (KAP) related to weight management among overweight or obese patients.

**Methods:**

A cross-sectional study was conducted in May 2024 at the First Hospital of Zhangjiakou, focusing on overweight or obese patients. Self-administered questionnaires were used to collect demographic data and assess participants' KAP scores.

**Results:**

A total of 527 valid responses were obtained, with 299 (56.74%) respondents being female. The mean scores for knowledge, attitudes, and practices were 6.09 ± 2.93 (possible range: 0–11), 22.79 ± 3.02 (possible range: 8–40), and 32.89 ± 9.72 (possible range: 9–45), respectively. Correlation analysis revealed a significant positive relationship between knowledge and practices (*r* = 0.305, *P* < 0.001), and a negative relationship between attitudes and practices (*r* = −0.516, *P* < 0.001). Structural equation modeling showed that knowledge directly influenced attitudes (β = 0.897, *P* = 0.008), and attitudes directly influenced practices (β = 1.108, *P* = 0.008). Additionally, knowledge had an indirect effect on practices through attitudes (β = 0.994, *P* = 0.007).

**Conclusions:**

Overweight or obese patients demonstrated inadequate knowledge, negative attitudes, and proactive practices toward weight management. These findings highlight the need for targeted educational interventions to enhance weight management knowledge and foster positive attitudes, ultimately leading to improved health practices within this population.

## Introduction

Overweight and obesity have become significant global health concerns, affecting nearly 50% of adults in China, with a prevalence of 34.3% for overweight and 16.4% for obesity (1). Obesity is closely associated with a range of serious health conditions, including type 2 diabetes, cardiovascular diseases, and certain cancers, contributing to increased morbidity and mortality (2, 3). Enhancing the understanding of comprehensive obesity management is essential to alleviate the ongoing burden on healthcare systems.

Several factors contribute to the high prevalence of overweight and obesity. Urbanization and nutritional transitions in low- and middle-income countries have increased consumption of unhealthy, calorie-dense foods and reduced physical activity, worsening the obesity crisis (4). Cultural shifts toward sedentary lifestyles, driven by technological advances and urban work environments, further decrease physical activity levels. Economic factors, such as the availability of inexpensive, high-calorie foods, also play a role in rising obesity rates (5). Psychological factors like stress, depression, and anxiety are linked to emotional eating and poor dietary choices (6). Socio-demographic characteristics, including age, gender, and education, influence obesity prevalence, with middle-aged adults, women, and those with lower education being more susceptible (7). Additionally, limited healthcare access, particularly in rural areas, hinders early intervention (8). Genetic predisposition, though not modifiable, interacts with environmental factors to increase obesity risk.

Given obesity's multifactorial nature, a one-size-fits-all solution is challenging. Current management focuses on lifestyle modifications, particularly dietary changes and physical activity (5). However, recent research suggests that combining these interventions with behavioral counseling and regular follow-ups may improve adherence and outcomes (9).

The knowledge, attitudes, and practices (KAP) survey is a valuable tool for assessing a group's understanding, beliefs, and behaviors regarding health issues, based on the principle that knowledge shapes attitudes, which in turn influence behaviors (10, 11). This approach is particularly relevant for populations at risk for chronic conditions like type 2 diabetes, cardiovascular diseases, and metabolic disorders. KAP surveys help identify educational gaps and design tailored interventions that consider cultural and social contexts. Given the rising obesity prevalence, it is crucial to assess KAP to design interventions that promote healthier lifestyles and improve clinical outcomes. Previous KAP studies on obesity have examined university students in China (10) and Iranian mothers' attitudes toward childhood obesity (12). However, comprehensive KAP studies on obesity management remain limited, with most research targeting either the general population or specific subgroups, limiting generalizability.

Research focusing on overweight and obese patients seeking clinical care is essential, as this group faces the most pressing need for effective weight management interventions. Unlike general population studies, focusing on this specific group provides more accurate, clinically relevant insights into the barriers and facilitators of weight management. This targeted approach fills a critical gap in the literature, allowing for the development of more effective, tailored interventions for individuals actively managing obesity (13, 14).

Thus, this study aimed to investigate the KAP related to weight management among overweight or obese patients.

## Materials and methods

### Study design and participants

This cross-sectional study was conducted in May 2024 at the First Hospital of Zhangjiakou, focusing on overweight or obese patients. Ethical approval was granted by the Ethics Committee of the First Hospital of Zhangjiakou (Approval No.: 2022038), and informed consent was obtained from all participants.

Inclusion criteria: (1) age 18 years or older; (2) previously or currently meeting the diagnostic criteria for overweight and obesity, including body mass index (BMI) ≥24 kg/m^2^, waist circumference ≥90 cm for men and ≥85 cm for women, waist-to-hip ratio >0.90 for men or ≥0.85 for women, and body fat percentage >25% for men and >30% for women (15); (3) willingness to participate in the study.

Exclusion criteria: (1) patients with severe chronic diseases; (2) patients with cognitive or mental disorders that would impede their ability to cooperate with the study.

### Questionnaire

The questionnaire was developed with reference to the Expert Consensus on the Weight Management Process for Overweight and Obese Populations (2021) (16). A preliminary survey was conducted with 23 valid responses, and a reliability test yielded a Cronbach's alpha of 0.847, indicating acceptable internal consistency.

The final questionnaire, administered in Chinese, consisted of four sections: demographic data, knowledge, attitude, and practice dimensions. The demographic section gathered basic information, including variables such as gender, age, residence, educational background, occupation, average monthly income, duration of being overweight or obese, current BMI, diet in the past year, and the presence of obesity-related conditions (e.g., high blood lipids, high blood sugar, high uric acid, high blood pressure, or other related diseases). The knowledge section comprised 11 items, with 1 point awarded for correct answers and 0 points for incorrect or unclear responses, resulting in a total score range of 0–11. The attitude section included 8 questions. Items 2 and 5–8 were positively worded and scored on a 5-point Likert scale, with “strongly agree” assigned 5 points and “strongly disagree” assigned 1 point. In contrast, items 1, 3, and 4 were negatively worded and reverse scored, with “strongly agree” assigned 1 point and “strongly disagree” assigned 5 points, yielding a total score range of 8–40. Reflecting incorrect or misleading beliefs and were therefore reverse scored to ensure consistent directionality with positively framed items. Reverse-coding was applied during data analysis to maintain the internal reliability of the constructs. The practice section consisted of 9 questions, scored on a 5-point Likert scale ranging from 1 (never) to 5 (always), with a total score range of 9–45. A score above 70% of the maximum in each section was considered indicative of adequate knowledge, positive attitudes, and proactive practices (17, 18).

### Questionnaire distribution and quality control

The questionnaires were distributed through both online and offline methods. For offline distribution, the questionnaire link was shared with overweight or obese outpatients via WeChat, and participants were informed about the study's purpose, with informed consent obtained. For online distribution, the questionnaire link was sent to participants in the WeChat weight loss group organized by the Department of Nutrition. The researchers explained the questionnaire's purpose and provided instructions for completion. It was ensured that all questions were mandatory and that each participant could submit only one response per IP address. Responses with illogical or overly consistent answer patterns were deemed invalid.

### Sample size calculations

The sample size was calculated using the formula for cross-sectional studies (19): α = 0.05, $n={\left(\frac{Z_{1-\alpha /2}}{\delta }\right)}^{2}\times P\times \left(1-P\right)$ where *Z*_1−α/2_ = 1.96 when α = 0.05, The assumed degree of variability (*P* = 0.5) was used to maximize the required sample size, and δ (admissible error) was set at 5%. The theoretical sample size was calculated to be 480, with an additional 20% included to account for potential dropouts.

### Statistical methods

Data analysis was conducted using SPSS 27.0 and AMOS 26.0 (IBM, Armonk, NY, USA). Continuous variables are presented as means and standard deviations (SD), while categorical data are expressed as frequency (*n*) and percentage (%). Normality tests were performed for continuous data, with *t*-tests or ANOVA used for normally distributed variables. Non-parametric tests (Mann-Whitney *U* test or Kruskal-Wallis test) were employed for non-normally distributed variables. Spearman correlation analysis was conducted to examine the relationships between knowledge, attitude, and practice scores. Structural equation modeling (SEM) was employed to test the following hypotheses: (1) knowledge impacts attitudes; (2) knowledge impacts practices both directly and indirectly through attitudes; and (3) attitudes impact practices. The model fit was evaluated using several indices, including Root Mean Square Error of Approximation (RMSEA), Standardized Root Mean Square Residual (SRMR), Incremental Fit Index (IFI), Tucker–Lewis Index (TLI), and Comparative Fit Index (CFI). The initial model was pre-specified based on theory. Modification indices (MI) provided by AMOS were examined during model refinement to identify correlated errors or potential paths that could improve model fit. Pairs of items with the highest MI values were sequentially adjusted, applying one modification at a time, with re-estimation after each adjustment. No *post-hoc* modifications were made beyond those supported by MI results. Univariate logistic regression was first performed to identify factors associated with proactive practices (defined as a practice score ≥70% of the total possible score). Variables with *P* < 0.05 in the univariate analysis were subsequently included in the multivariate logistic regression model to determine independent variables. A two-sided *P*-value of < 0.05 was considered statistically significant.

## Results

### Basic characteristics

A total of 527 valid responses were collected. To assess construct validity, confirmatory factor analysis (CFA) was conducted (Supplementary Figure S1, Supplementary Table S1) and demonstrated an overall acceptable model fit (CMIN/DF = 4.951, RMSEA = 0.087, IFI = 0.865, TLI = 0.852, CFI = 0.865). These analyses supported the unidimensional structure assumed in the SEM model. Among the respondents, 299 (56.74%) were female, 316 (59.96%) were under 38 years of age, 258 (48.96%) lived in urban areas, 305 (57.87%) held an associate's or bachelor's degree, 192 (36.43%) were employed in medical-related fields, and 453 (85.96%) had obesity-related conditions. Additionally, 90 participants (17.08%) had a BMI of < 24 kg/m^2^, indicating that they were previously diagnosed as overweight or obese but have since maintained a normal BMI. The mean scores for knowledge, attitudes, and practices were 6.09 ± 2.93 (possible range: 0–11), 22.79 ± 3.02 (possible range: 8–40), and 32.89 ± 9.72 (possible range: 9–45), respectively. Significant differences in knowledge, attitudes, and practices scores were observed based on education level (*P* = 0.006, *P* < 0.001, *P* = 0.004), occupation (*P* < 0.001 for all), average monthly income (*P* < 0.001 for all), current BMI (*P* < 0.001, *P* < 0.001, *P* = 0.021), and obesity-related conditions (*P* < 0.001 for all). Additionally, knowledge and attitudes scores varied significantly by gender (*P* < 0.001 and *P* = 0.042), age (*P* = 0.009 and *P* = 0.005), residence (*P* < 0.001 for both), duration of being overweight/obese (*P* = 0.004 and *P* = 0.029), and diet type (*P* < 0.001 for both) (Table 1). Among respondents, 188 (35.67%) reported improvement in high blood sugar, 171 (32.45%) in high cholesterol, 125 (23.72%) in high uric acid, and 86 (16.32%) in high blood pressure. Only 3 (0.57%) mentioned improvement in other unspecified conditions, while 6 (1.14%) reported no change after weight loss.

**Table 1:** Basic information.

**Variables**	***N* (%)**	**Knowledge, mean ±SD**	***P***	**Attitudes, mean ±SD**	***P***	**Practices, mean ±SD**	***P***
***N*** **=** **527**		6.09 ± 2.93		22.79 ± 3.02		32.89 ± 9.72	
**Gender**			**< 0.001**		**0.042**		0.06
Male	228 (43.26)	5.62 ± 2.87		22.45 ± 2.90		33.32 ± 9.91	
Female	299 (56.74)	6.44 ± 2.93		23.04 ± 3.10		32.57 ± 9.57	
**Age (median: 38 years)**			**0.009**		**0.005**		0.153
Under 38 years old	316 (59.96)	5.78 ± 3.11		23.11 ± 3.09		31.96 ± 10.44	
38 years old and above	211 (40.04)	6.55 ± 2.56		22.30 ± 2.86		34.30 ± 8.35	
**Residence**			**< 0.001**		**< 0.001**		0.206
Rural	138 (26.19)	5.73 ± 2.46		21.99 ± 2.59		33.95 ± 9.10	
Urban	258 (48.96)	6.64 ± 3.21		23.55 ± 3.31		32.02 ± 10.02	
Suburban	131 (24.86)	5.37 ± 2.59		22.12 ± 2.45		33.50 ± 9.65	
**Education**			**0.006**		**< 0.001**		**0.004**
Middle school or below	99 (18.79)	5.94 ± 2.28		21.54 ± 2.56		34.23 ± 8.63	
High school/technical school	63 (11.95)	6.24 ± 2.28		21.86 ± 2.66		34.84 ± 8.91	
Associate degree/bachelor's degree	305 (57.87)	5.85 ± 3.28		23.39 ± 3.10		31.34 ± 10.52	
Master's degree or above	60 (11.39)	7.38 ± 2.21		22.75 ± 2.88		36.55 ± 5.59	
**Occupation**			**< 0.001**		**< 0.001**		**< 0.001**
Medical student/doctor/nurse/other medical-related work	192 (36.43)	6.41 ± 3.30		23.61 ± 3.12		31.49 ± 10.18	
Non-medical-related work	258 (48.96)	6.49 ± 2.18		21.66 ± 2.53		36.23 ± 6.93	
Unemployed/non-medical student	77 (14.61)	3.94 ± 3.23		24.51 ± 2.86		25.21 ± 11.33	
**Average monthly income, CNY**			**< 0.001**		**< 0.001**		**< 0.001**
< 5,000	239 (45.35)	5.12 ± 3.37		23.69 ± 2.98		28.85 ± 11.11	
5,000–10,000	158 (29.98)	7.24 ± 2.58		22.78 ± 3.09		34.80 ± 8.39	
10,000–20,000	68 (12.9)	6.50 ± 1.70		21.06 ± 2.11		37.87 ± 2.59	
>20,000	62 (11.76)	6.44 ± 1.37		21.19 ± 2.36		38.18 ± 3.83	
**Period of overweight/obese**			**0.003**		**0.029**		0.982
< 3 years	138 (26.19)	6.80 ± 3.23		23.46 ± 3.24		32.67 ± 9.87	
3–5 years	145 (27.51)	5.92 ± 2.80		22.58 ± 3.09		33.14 ± 9.69	
6–10 years	136 (25.81)	5.70 ± 2.68		22.37 ± 2.71		32.99 ± 9.62	
>10 years	108 (20.49)	5.90 ± 2.87		22.72 ± 2.91		32.74 ± 9.81	
**Current BMI**			**< 0.001**		**< 0.001**		**0.021**
BMI < 24.0 kg/m^2^	90 (17.08)	6.87 ± 3.35		24.24 ± 3.15		31.19 ± 9.55	
24.0 kg/m^2^ ≤ BMI < 28.0 kg/m^2^	100 (18.98)	6.90 ± 3.11		23.32 ± 3.26		32.03 ± 9.77	
28.0 kg/m^2^ ≤ BMI < 32.5 kg/m^2^	117 (22.2)	6.02 ± 3.04		22.47 ± 3.34		33.15 ± 9.86	
32.5 kg/m^2^ ≤ BMI < 37.5 kg/m^2^	137 (26)	5.36 ± 2.40		22.26 ± 2.49		33.42 ± 9.88	
BMI ≥ 37.5 kg/m^2^	83 (15.75)	5.55 ± 2.44		21.87 ± 2.19		34.54 ± 9.20	
**Diet in the past year**			**< 0.001**		**< 0.001**		0.84
High-protein diet	104 (19.73)	5.73 ± 3.21		22.80 ± 2.86		32.58 ± 10.16	
Calorie-restricted diet	145 (27.51)	5.92 ± 2.74		22.52 ± 2.91		33.50 ± 9.18	
Intermittent fasting	90 (17.08)	5.82 ± 2.68		22.46 ± 3.04		32.76 ± 9.71	
Other weight-loss diets	95 (18.03)	5.42 ± 2.58		22.27 ± 2.75		33.36 ± 9.92	
Have not followed any weight-loss diet	93 (17.65)	7.68 ± 2.95		24.03 ± 3.33		31.96 ± 9.93	
**Presence of obesity-related conditions** ^*^			**< 0.001**		**< 0.001**		**< 0.001**
Yes	453 (85.96)	5.58 ± 2.68		22.24 ± 2.68		33.36 ± 9.78	
No	74 (14.04)	9.20 ± 2.43		26.15 ± 2.82		30.07 ± 8.88	

^*^Obesity-related conditions: high blood lipids, high blood sugar, high uric acid, high blood pressure or other obesity-related diseases.

### Distribution of responses in knowledge, attitudes, and practices

In the knowledge dimension, the three items most frequently marked as “Unclear” were: “Weight management should consider personal health conditions and preferences, with gradual progression in exercise choices” (K7), chosen by 68.69%; “Dietary recommendations for weight management include strictly controlling oil and fat intake, moderately limiting refined rice, flour, and meat, and ensuring sufficient intake of vegetables, fruits, and dairy products” (K6), chosen by 67.55%; and “Behavior modification recommendations for weight management include daily tracking of weight, diet, and exercise, as well as regular waist and hip circumference measurements” (K8), also chosen by 67.55% (Table 2).

**Table 2:** Knowledge dimension.

**Items**	**Correctness rate (%)**
1. Obesity/overweight is a disease.	346 (65.65)
2. The causes of overweight/obesity include genetic and environmental factors.	337 (63.95)
3. Obesity/overweight can lead to complications such as type 2 diabetes, dyslipidemia, hypertension, coronary atherosclerotic heart disease, non-alcoholic fatty liver disease, polycystic ovary syndrome, female infertility, sleep apnea syndrome, osteoarthritis, and gout.	349 (66.22)
4. Lifestyle interventions for obesity/overweight patients mainly include diet, exercise, and behavior modification.	354 (67.17)
5. It is not necessary to supplement micronutrients during medical nutrition weight loss.	189 (35.86)
6. Dietary recommendations for weight management include strictly controlling the intake of oils and fats, moderately limiting refined rice, flour, and meat, and ensuring sufficient intake of vegetables, fruits, and dairy products.	356 (67.55)
7. Weight management should take into account personal health conditions and preferences, with a gradual progression in exercise choices.	362 (68.69)
8. Behavior modification recommendations for weight management include daily recording of weight, diet, and exercise, as well as regular measurement of waist and hip circumference.	356 (67.55)
9. Behavioral interventions for weight management do not include maintaining a regular sleep schedule.	182 (34.54)
10. Surgery alone can cure obesity/overweight.	200 (37.95)
11. The clinical goals of weight management for overweight/obese patients do not include preventing obesity-related diseases.	177 (33.59)

In the attitudes dimension, 26.38% strongly agreed, and 35.1% agreed that reducing food intake alone would lead to weight loss (A3), while 27.32% strongly agreed, and 28.65% agreed that there was no need to adjust their diet, exercise, or behavioral habits if they were taking weight-loss medications (A4). In contrast, 13.28% were very reluctant to undergo weight loss surgery (A7), and 11.57% were very reluctant to use weight loss medications (A8) (Table 3).

**Table 3:** Attitude dimension.

**Items**	***N*** **(%)**				
	**Strongly agree**	**Agree**	**Neutral**	**Disagree**	**Strongly disagree**
1. I believe that obesity/overweight has severely affected my life.	307 (58.25)	121 (22.96)	92 (17.46)		7 (1.33)
2. I believe that weight management is important.	187 (35.48)	211 (40.04)	47 (8.92)	39 (7.4)	43 (8.16)
3. I believe that as long as I eat less, my weight will decrease.	139 (26.38)	185 (35.1)	58 (11.01)	93 (17.65)	52 (9.87)
4. I believe that if I take weight-loss medication, there is no need to adjust my diet, exercise, or behavior habits.	144 (27.32)	151 (28.65)	52 (9.87)	104 (19.73)	76 (14.42)
5. I believe that medical weight loss helps prevent weight regain after losing weight.	188 (35.67)	192 (36.43)	60 (11.39)	36 (6.83)	51 (9.68)
6. I am willing to try telemedicine for medical nutrition weight loss.	177 (33.59)	196 (37.19)	64 (12.14)	42 (7.97)	48 (9.11)
7. I am willing to try weight-loss surgery.	128 (24.29)	158 (29.98)	76 (14.42)	95 (18.03)	70 (13.28)
8. I am willing to try weight-loss medication.	145 (27.51)	158 (29.98)	68 (12.9)	95 (18.03)	61 (11.57)

Regarding practices, 11.57% never followed up with a doctor for weight loss guidance (P6), 10.63% never appropriately controlled their intake of carbohydrates and fats, and 10.44% never maintained regular exercise after successful weight loss (P3) (Table 4).

**Table 4:** Practice dimension.

**Items**	***N*** **(%)**				
	**Always**	**Often**	**Sometimes**	**Occasionally**	**Never**
1. I control my intake of carbohydrates and fats appropriately.	141 (26.76)	192 (36.43)	90 (17.08)	48 (9.11)	56 (10.63)
2. I follow the guidance of doctors or professionals to prepare weight-loss meals.	168 (31.88)	163 (30.93)	82 (15.56)	60 (11.39)	54 (10.25)
3. After scientific weight loss, I maintain regular exercise.	150 (28.46)	181 (34.35)	81 (15.37)	60 (11.39)	55 (10.44)
4. After scientific weight loss, I continue to follow medical advice for long-term lifestyle interventions.	160 (30.36)	182 (34.54)	82 (15.56)	56 (10.63)	47 (8.92)
5. I actively seek encouragement and support from family and social circles and, if necessary, accept professional weight-loss education and guidance.	171 (32.45)	174 (33.02)	79 (14.99)	59 (11.2)	44 (8.35)
6. I record my weight, diet, and exercise daily.	153 (29.03)	187 (35.48)	72 (13.66)	66 (12.52)	49 (9.3)
7. I maintain a healthy and positive mindset during medical weight loss.	183 (34.72)	181 (34.35)	68 (12.9)	42 (7.97)	53 (10.06)
8. I proactively seek knowledge about weight management.	159 (30.17)	191 (36.24)	80 (15.18)	57 (10.82)	40 (7.59)
9. I regularly follow up with my doctor and lose weight under their guidance.	164 (31.12)	175 (33.21)	75 (14.23)	52 (9.87)	61 (11.57)

### Correlations between knowledge, attitudes, and practices

Correlation analysis revealed a significant positive relationship between knowledge and practices (*r* = 0.305, *P* < 0.001), and a significant negative relationship between attitudes and practices (*r* = −0.516, *P* < 0.001). However, the correlation between knowledge and attitudes was not statistically significant (*r* = 0.027, *P* = 0.533) (Supplementary Table S2).

### Structural equation modeling

**Figure 1:**
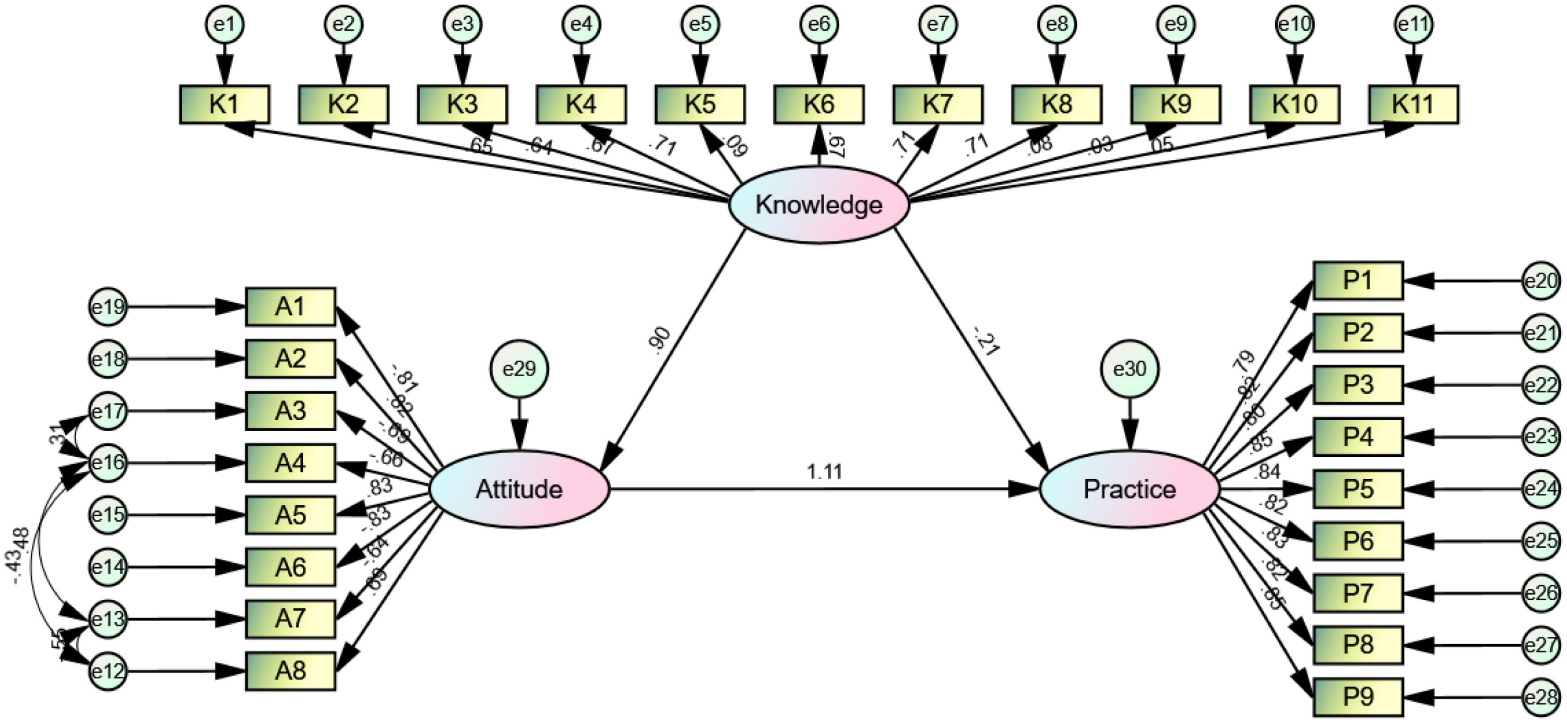
Structural equation model.

The structural model demonstrated an acceptable fit (CMIN/DF = 3.843, RMSEA = 0.074; IFI = 0.904; TLI = 0.894; CFI = 0.904), indicating good model-data fit (Supplementary Table S3). Results showed that knowledge directly influenced attitudes (β = 0.897, *P* = 0.008), and attitudes directly influenced practices (β = 1.108, *P* = 0.008). Additionally, knowledge had an indirect effect on practices through attitudes (β = 0.994, *P* = 0.007) (Table 5 and Figure 1).

**Table 5:** Mediating effect values.

**Model paths**	**Standardized total effects**			**Standardized direct effects**			**Standardized indirect effects**		
	β **(95% CI)**	**SE**	***P***	β **(95% CI)**	**SE**	***P***	β **(95% CI)**	**SE**	***P***
Knowledge → Attitude	0.897 (0.847–0.938)	0.023	0.008	0.897 (0.847–0.938)	0.023	0.008			
Knowledge → Practice	0.782 (0.686–0.839)	0.035	0.020	−0.212 (−0.596–0.055)	0.161	0.184			
Attitude → Practice	1.108 (0.857–1.486)	0.153	0.008	1.108 (0.857–1.486)	0.153	0.008			
Knowledge → Practice							0.994 (0.750–1.378)	0.155	0.007

### Multivariate logistic regression associated with practices

Multivariate logistic regression showed that knowledge (OR = 2.037, 95% CI: 1.723–2.409, *P* < 0.001), attitudes (OR = 0.567, 95% CI: 0.485–0.663, *P* < 0.001), having a master's degree or above (OR = 5.308, 95% CI: 1.182–23.845, *P* = 0.029), monthly income of 10,000–20,000 CNY (OR = 6.724, 95% CI: 1.233–36.652, *P* = 0.028), monthly income >20,000 CNY (OR = 11.151, 95% CI: 1.616–76.921, *P* = 0.014), having followed other weight-loss diets (OR = 3.579, 95% CI: 1.170–10.943, *P* = 0.025), and the presence of obesity-related conditions (OR = 0.096, 95% CI: 0.033–0.282, *P* < 0.001) were significantly associated with proactive practices (Supplementary Table S4).

## Discussion

Overweight and obese patients demonstrated insufficient knowledge, negative attitudes, and inconsistent practices regarding weight management. Interventions aimed at enhancing patient education on weight management could improve attitudes and foster more effective practices, ultimately contributing to better health outcomes.

These observations are consistent with growing research that identifies inadequate health literacy and negative attitudes as major barriers to effective obesity management. For example, similar studies have reported that patients with low levels of knowledge about the causes and consequences of obesity are more likely to struggle with weight management, leading to poor adherence to dietary recommendations and exercise regimens (20, 21). This lack of awareness and understanding contributes to the persistence of obesity-related conditions, despite the availability of various weight management strategies. It's evident that inadequate knowledge and negative attitudes toward weight management are common, but the reasons for these deficits may differ based on cultural, socioeconomic, and environmental factors. For instance, a study in a different population found that, despite widespread awareness of the health risks associated with obesity, knowledge about practical steps for weight loss was often lacking (22, 23). This mirrors our findings where certain critical aspects of weight management, such as micronutrient supplementation and regular sleep schedules, were poorly understood. These knowledge gaps could explain why obesity-related conditions remain prevalent and why patient adherence to weight management programs remains suboptimal.

In terms of the relationship between knowledge, attitudes, and practices, our correlation analysis and SEM provide further insight. We found that knowledge positively correlated with practices, which aligns with prior research showing that patients who possess greater knowledge about weight management are more likely to engage in healthy behaviors (24, 25). However, the negative correlation between attitudes and practices in our study is particularly noteworthy, as it suggests that even when patients understand the importance of weight management, negative attitudes may impede their ability to translate this knowledge into action. This finding is supported by previous research that has highlighted the critical role of attitudes in shaping health behaviors. For instance, studies have shown that patients who hold negative views about their ability to lose weight are less likely to adhere to dietary and exercise plans, even when they are knowledgeable about the benefits (26, 27). This reinforces the importance of addressing not just knowledge gaps but also attitudinal barriers in weight management interventions. Interestingly, while correlation analysis indicated a negative relationship between attitudes and practices, SEM revealed a positive direct effect. This discrepancy may stem from methodological differences: correlation analysis captures simple pairwise associations, whereas SEM accounts for latent variables and mediating pathways, offering a more nuanced interpretation of relationships among constructs. Additionally, potential measurement issues such as item phrasing, especially the inclusion of reverse-scored attitude items, may have influenced the observed negative correlation. Furthermore, reverse causality whereby poor weight management practices lead to frustration and negative attitudes cannot be ruled out in a cross-sectional design. This warrants further exploration in future longitudinal studies. This difference can be attributed to the distinct analytical methods: while correlation analysis captures direct relationships, SEM accounts for both direct and indirect effects, incorporating potential mediators. This suggests that knowledge may indirectly enhance practices through attitudes, which aligns with SEM findings. Additionally, the correlation analysis did not show a significant relationship between knowledge and attitudes, in contrast to SEM results. This discrepancy may be due to unobserved factors, such as self-efficacy or motivation, that influence the link between knowledge and attitudes.

The significant differences in KAP across demographic variables also warrant discussion, as they shed light on potential disparities in weight management outcomes. Gender differences revealed that women had higher knowledge and attitude scores than men, yet no significant difference was observed in practice scores. This discrepancy may reflect the fact that women are often more exposed to health information and societal pressures regarding weight, yet they may face additional psychological or environmental barriers that prevent them from consistently applying this knowledge in practices (28, 29). Studies have suggested that women, particularly those in certain cultural contexts, may experience higher levels of body dissatisfaction and anxiety about weight loss, which can paradoxically hinder their ability to adopt and maintain healthy practices (30, 31). In contrast, men may be less informed about weight management but more likely to engage in physical activities due to different social norms and expectations around weight and fitness.

Age also played a significant role, with older participants demonstrating higher knowledge and practice scores compared to their younger counterparts, although their attitude scores were lower. This could be attributed to older individuals having more experience with weight management, either through personal efforts or interactions with healthcare providers. However, their lower attitude scores may suggest that over time, they have become more disillusioned with the efficacy of weight management strategies, possibly due to repeated failures or frustrations with their results. This finding aligns with research showing that older adults often become more resigned to their health conditions, which can negatively affect their motivation to engage in proactive health behaviors (32, 33). On the other hand, younger participants may have a more positive outlook but lack the practical experience or knowledge needed to effectively manage their weight.

Residence was another significant factor, with urban residents showing higher knowledge and attitude scores than rural or suburban residents. This difference is likely due to greater access to health information, resources, and services in urban areas. Previous studies have demonstrated that rural populations often have limited access to healthcare services and weight management programs, which contributes to lower levels of health literacy and poorer health outcomes (34, 35). In this context, the findings highlight the need for targeted interventions in rural and suburban areas to improve awareness and attitudes toward weight management.

Education level was closely linked to KAP scores, with higher education levels corresponding to better knowledge and practice scores. However, interestingly, participants with higher education levels did not have significantly better attitude scores. This suggests that while education equips individuals with the knowledge needed to manage their weight, it does not necessarily translate into more positive attitudes. These descriptive patterns were further examined and partially confirmed through multivariate regression, which adjusted for potential confounders, reinforcing the importance of socioeconomic factors in shaping proactive weight management behaviors. This could be due to the fact that individuals with higher education levels may have a more nuanced understanding of the challenges associated with weight management, which may make them more critical or skeptical about the effectiveness of various strategies (36, 37).

In the knowledge dimension, a substantial proportion of respondents were unclear about key concepts such as the role of micronutrient supplementation and the importance of maintaining regular sleep schedules. These findings are consistent with previous studies showing that many patients lack a comprehensive understanding of the factors that contribute to successful weight loss beyond diet and exercise (38, 39). This suggests that current educational programs may be too narrow in focus and fail to address the broader aspects of weight management. Similarly, negative attitudes toward weight-loss surgery and medication were prevalent in our sample, with many respondents expressing reluctance to consider these options. Addressing these misconceptions through targeted education and counseling could help improve patient acceptance of these effective treatment options.

In terms of practical recommendations, several targeted interventions can be suggested. First, educational programs should expand beyond the traditional focus on diet and exercise to include topics such as micronutrient supplementation, the importance of regular sleep, and behavioral strategies for sustaining weight loss. These programs could be delivered through tailored health education sessions in community health centers, mobile health applications that use simple language and visuals, and village-level outreach led by trained local health workers. For rural or less-educated subgroups, using culturally appropriate audio-visual materials and interactive activities may enhance comprehension and engagement. Telemedicine services could also be expanded in these regions to improve access to professional guidance. Second, healthcare providers should adopt motivational interviewing and other behavioral counseling techniques to address negative attitudes and promote more positive perspectives on weight management. This approach has been shown to be effective in helping patients overcome resistance to lifestyle changes and improve adherence to weight management programs (40). Additionally, peer support groups or mentorship programs could be implemented to provide ongoing encouragement and accountability, particularly for patients who struggle with maintaining consistent practices. Studies have shown that social support is a key factor in long-term weight loss success, particularly for women and individuals from lower socioeconomic backgrounds (41, 42).

Given the differences in KAP scores across demographic variables such as gender, residence, and education, it is important to tailor interventions to meet the specific needs of these subgroups. For example, for rural residents or those with lower education levels, mobile health platforms or community-based education programs may be effective in improving knowledge and practices. For individuals who express reluctance toward medical interventions such as surgery or medication, providing counseling and information about the risks and benefits of these options, as well as connecting them with patients who have successfully undergone these treatments, may help reduce anxiety and increase acceptance. These strategies should be informed by evidence-based research and continuously evaluated to ensure they effectively address the barriers faced by each subgroup (43, 44).

This study has several limitations. First, the cross-sectional design prevents the establishment of causal relationships between knowledge, attitudes, and practices. Second, the use of self-reported questionnaires may introduce response bias, as participants may overestimate or underestimate their behaviors. Additionally, the study was conducted at a single tertiary hospital in Zhangjiakou, a mid-sized city in northern China, which may limit the generalizability of the findings. However, the hospital serves a relatively diverse patient population from both urban and rural settings, providing a degree of variation in socioeconomic and educational backgrounds. Nonetheless, caution should be exercised when extending these findings to other regions in China or internationally, where cultural, healthcare access, and lifestyle factors may differ significantly. The questionnaire also did not assess psychological factors such as motivation, self-efficacy, or health literacy, which may mediate or moderate the observed relationships among KAP components. The absence of these variables may limit the explanatory power of our model, particularly regarding the mechanism by which knowledge influences behavior. Future research will aim to incorporate validated measures of self-efficacy and motivational readiness to provide a more comprehensive understanding of behavioral determinants in weight management. Social desirability bias may have further influenced participants' responses, particularly regarding practices. Moreover, the study relied on a predefined cutoff (70% of the maximum score) to define adequacy in KAP, which may not fully reflect clinical significance or patient-specific needs. Lastly, although a 70% threshold was used to define adequacy in the KAP dimensions, the choice of this cutoff may influence the interpretation of adequacy, and its validity should be further examined in future studies.

Despite these limitations, this study has several strengths. It included a relatively large and diverse sample, enhancing the reliability and representativeness of the findings. The use of structural equation modeling allowed for a comprehensive examination of direct and indirect relationships among knowledge, attitudes, and practices, offering valuable insights into the mechanisms underlying weight management behavior. Moreover, the questionnaire demonstrated good internal consistency and was based on national expert consensus guidelines, supporting its relevance and credibility.

In conclusion, overweight or obese patients demonstrated inadequate knowledge, negative attitudes, and limited proactive practices regarding weight management. These findings underscore the importance of not only delivering educational content but also addressing underlying attitudinal barriers and behavioral patterns. Interventions tailored to gender, age, and residential context may enhance effectiveness. Additionally, integrating psychological support and community-based health promotion may bridge the gap between knowledge and practice. Future studies should explore longitudinal changes in KAP and investigate the role of psychosocial determinants such as motivation and self-efficacy. Expanding research across multiple centers and regions would also help validate and generalize these findings, especially among underrepresented or high-risk subgroups. At the policy level, integrating structured weight management education into national public health programs and promoting access to professional nutritional counseling, particularly in rural and underserved areas, may help address systemic gaps and support long-term obesity control efforts.

## Data Availability

The original contributions presented in the study are included in the article/Supplementary material, further inquiries can be directed to the corresponding authors.
