# Supplementary material for: Knowledge, attitudes, and practices regarding body weight management among patients with overweight or obesity: a cross-sectional study
Source: Front Public Health. 2025 Jul 23;13:1615478. doi: 10.3389/fpubh.2025.1615478 (PMC12325174; doi:10.3389/fpubh.2025.1615478)
Supplement: Supplementary file 1 [file Table_1.docx]

**Supplementary table 1 Confirmatory factor analysis**

|  |  |  | **Estimate** | **Standardized Estimate** | **S.E.** | **C.R.** | **P** |
| --- | --- | --- | --- | --- | --- | --- | --- |
| K1 | <--- | Knowledge | 1.000 | 0.649 |  |  |  |
| K2 | <--- | Knowledge | 0.997 | 0.639 | 0.078 | 12.712 | <0.001 |
| K3 | <--- | Knowledge | 1.035 | 0.674 | 0.078 | 13.280 | <0.001 |
| K4 | <--- | Knowledge | 1.084 | 0.711 | 0.078 | 13.876 | <0.001 |
| K5 | <--- | Knowledge | 0.139 | 0.089 | 0.072 | 1.925 | 0.054 |
| K6 | <--- | Knowledge | 1.030 | 0.677 | 0.077 | 13.340 | <0.001 |
| K7 | <--- | Knowledge | 1.064 | 0.706 | 0.077 | 13.805 | <0.001 |
| K8 | <--- | Knowledge | 1.088 | 0.716 | 0.078 | 13.950 | <0.001 |
| K9 | <--- | Knowledge | 0.135 | 0.088 | 0.072 | 1.892 | 0.059 |
| K10 | <--- | Knowledge | 0.053 | 0.034 | 0.073 | 0.725 | 0.468 |
| K11 | <--- | Knowledge | 0.072 | 0.047 | 0.071 | 1.019 | 0.308 |
| A1 | <--- | Attitude | 1.000 | 0.831 |  |  |  |
| A2 | <--- | Attitude | -1.294 | -0.767 | 0.063 | -20.674 | <0.001 |
| A3 | <--- | Attitude | 1.370 | 0.747 | 0.069 | 19.909 | <0.001 |
| A4 | <--- | Attitude | 1.469 | 0.737 | 0.075 | 19.543 | <0.001 |
| A5 | <--- | Attitude | -1.400 | -0.797 | 0.064 | -21.893 | <0.001 |
| A6 | <--- | Attitude | 1.398 | 0.804 | 0.063 | 22.192 | <0.001 |
| A7 | <--- | Attitude | 1.387 | 0.726 | 0.072 | 19.134 | <0.001 |
| A8 | <--- | Attitude | -1.447 | -0.761 | 0.071 | -20.448 | <0.001 |
| P1 | <--- | Practice | 1.000 | 0.794 |  |  |  |
| P2 | <--- | Practice | 1.068 | 0.818 | 0.050 | 21.544 | <0.001 |
| P3 | <--- | Practice | 1.035 | 0.805 | 0.049 | 21.059 | <0.001 |
| P4 | <--- | Practice | 1.070 | 0.854 | 0.047 | 22.874 | <0.001 |
| P5 | <--- | Practice | 1.047 | 0.834 | 0.047 | 22.116 | <0.001 |
| P6 | <--- | Practice | 1.048 | 0.825 | 0.048 | 21.777 | <0.001 |
| P7 | <--- | Practice | 1.063 | 0.832 | 0.048 | 22.040 | <0.001 |
| P8 | <--- | Practice | 0.987 | 0.813 | 0.046 | 21.361 | <0.001 |
| P9 | <--- | Practice | 1.122 | 0.851 | 0.049 | 22.741 | <0.001 |

**Supplementary table 2 Correlation analysis**

|  | Knowledge | Attitudes | Practices |
| --- | --- | --- | --- |
| Knowledge | 1 |  |  |
| Attitudes | 0.027 (P=0.533) | 1 |  |
| Practices | 0.305 (**P<0.001**) | -0.516 (**P<0.001**) | 1 |

**Supplementary table 3 Path analysis model fit**

| **Model fit indices** | **Ref.** | **Measured results** |
| --- | --- | --- |
| **CMIN/DF** | 1-3 excellent, 3-5 good | 3.843 |
| **RMSEA** | <0.08 good | 0.074 |
| **IFI** | >0.8 good | 0.904 |
| **TLI** | >0.8 good | 0.894 |
| **CFI** | >0.8 good | 0.904 |

Abbreviation:

CMIN/DF: Chi-square divided by degrees of freedom

RMSEA: Root Mean Square Error of Approximation

IFI: Incremental Fit Index

TLI: Tucker-Lewis Index

CFI: Comparative Fit Index

**Supplementary table 4 Univariate and multivariate logistic regression associated with practices**

|  | Univariate logistic regression | | Multivariate logistic regression | |
| --- | --- | --- | --- | --- |
|  | OR (95% CI) | P | OR (95% CI) | P |
| **Knowledge** | 1.404 (1.299-1.518) | <0.001 | 2.037 (1.723-2.409) | <0.001 |
| **Attitude** | 0.584 (0.530-0.644) | <0.001 | 0.567 (0.485-0.663) | <0.001 |
| **Gender** |  |  |  |  |
| Male | 1.242 (0.843-1.830) | 0.273 |  |  |
| Female | ref |  |  |  |
| **Age (median: 38 years)** |  |  |  |  |
| Under 38 years old | 0.569 (0.379-0.853) | 0.006 | 1.707 (0.774-3.763) | 0.185 |
| 38 years old and above | ref |  | ref |  |
| **Residence** |  |  |  |  |
| Rural | ref |  | ref |  |
| Urban | 0.475 (0.292-0.774) | 0.003 | 0.443 (0.179-1.098) | 0.079 |
| Suburban | 0.895 (0.499-1.607) | 0.711 | 1.236 (0.428-3.573) | 0.695 |
| **Education** |  |  |  |  |
| Middle school or below | ref |  | ref |  |
| High school/technical school | 1.197 (0.530-2.703) | 0.666 | 1.749 (0.411-7.432) | 0.449 |
| Associate degree/bachelor’s degree | 0.468 (0.272-0.807) | 0.006 | 3.147 (0.954-10.382) | 0.060 |
| Master’s degree or above | 1.435 (0.606-3.397) | 0.412 | 5.308 (1.182-23.845) | 0.029 |
| **Occupation** |  |  |  |  |
| Medical student/Doctor/Nurse/Other medical-related work | 2.768 (1.608-4.768) | <0.001 | 1.091 (0.416-2.863) | 0.859 |
| Non-medical-related work | 9.776 (5.468-17.478) | <0.001 | 1.834 (0.619-5.431) | 0.273 |
| Unemployed/Non-medical student | ref |  | ref |  |
| **Average monthly income, CNY** |  |  |  |  |
| <5000 | ref |  | ref |  |
| 5000-10000 | 3.230 (2.038-5.118) | <0.001 | 1.553 (0.677-3.562) | 0.299 |
| 10000-20000 | 28.140 (6.737-117.535) | <0.001 | 6.724 (1.233-36.652) | 0.028 |
| >20000 | 25.581 (6.112-107.067) | <0.001 | 11.151 (1.616-76.921) | 0.014 |
| **Period of overweight/obese** |  |  |  |  |
| <3 years | ref |  |  |  |
| 3-5 years | 1.321 (0.786-2.220) | 0.293 |  |  |
| 6-10 years | 1.257 (0.744-2.124) | 0.392 |  |  |
| >10 years | 1.124 (0.648-1.951) | 0.677 |  |  |
| **Current BMI** |  |  |  |  |
| BMI<24.0 kg/m^2^ | ref |  | ref |  |
| 24.0 kg/m^2^≤BMI<28.0 kg/m^2^ | 0.986 (0.546-1.779) | 0.962 | 0.415 (0.154-1.119) | 0.082 |
| 28.0 kg/m^2^≤BMI<32.5 kg/m^2^ | 1.538 (0.852-2.776) | 0.153 | 0.697 (0.236-2.055) | 0.512 |
| 32.5 kg/m^2^≤BMI<37.5 kg/m2 | 1.980 (1.101-3.559) | 0.022 | 1.270 (0.397-4.060) | 0.687 |
| BMI≥37.5 kg/m^2^ | 2.853 (1.393-5.844) | 0.004 | 1.687 (0.422-6.745) | 0.460 |
| **Diet in the past year** |  |  |  |  |
| High-protein diet | 1.489 (0.820-2.703) | 0.191 | 2.487 (0.868-7.128) | 0.090 |
| Calorie-restricted diet | 1.897 (1.077-3.341) | 0.027 | 1.822 (0.675-4.923) | 0.236 |
| Intermittent fasting | 1.659 (0.886-3.109) | 0.114 | 2.174 (0.704-6.710) | 0.177 |
| Other weight-loss diets | 1.889 (1.007-3.545) | 0.048 | 3.579 (1.170-10.943) | 0.025 |
| Have not followed any weight-loss diet | ref |  | ref |  |
| **Presence of obesity-related conditions^*^** |  |  |  |  |
| Yes | 0.246 (0.148-0.408) | <0.001 | 0.096 (0.033-0.282) | <0.001 |
| No | ref |  | ref |  |
