# Supplementary figures and images for: Knowledge, attitudes, and practices regarding body weight management among patients with overweight or obesity: a cross-sectional study
Source: Front Public Health. 2025 Jul 23;13:1615478. doi: 10.3389/fpubh.2025.1615478 (PMC12325174; doi:10.3389/fpubh.2025.1615478)

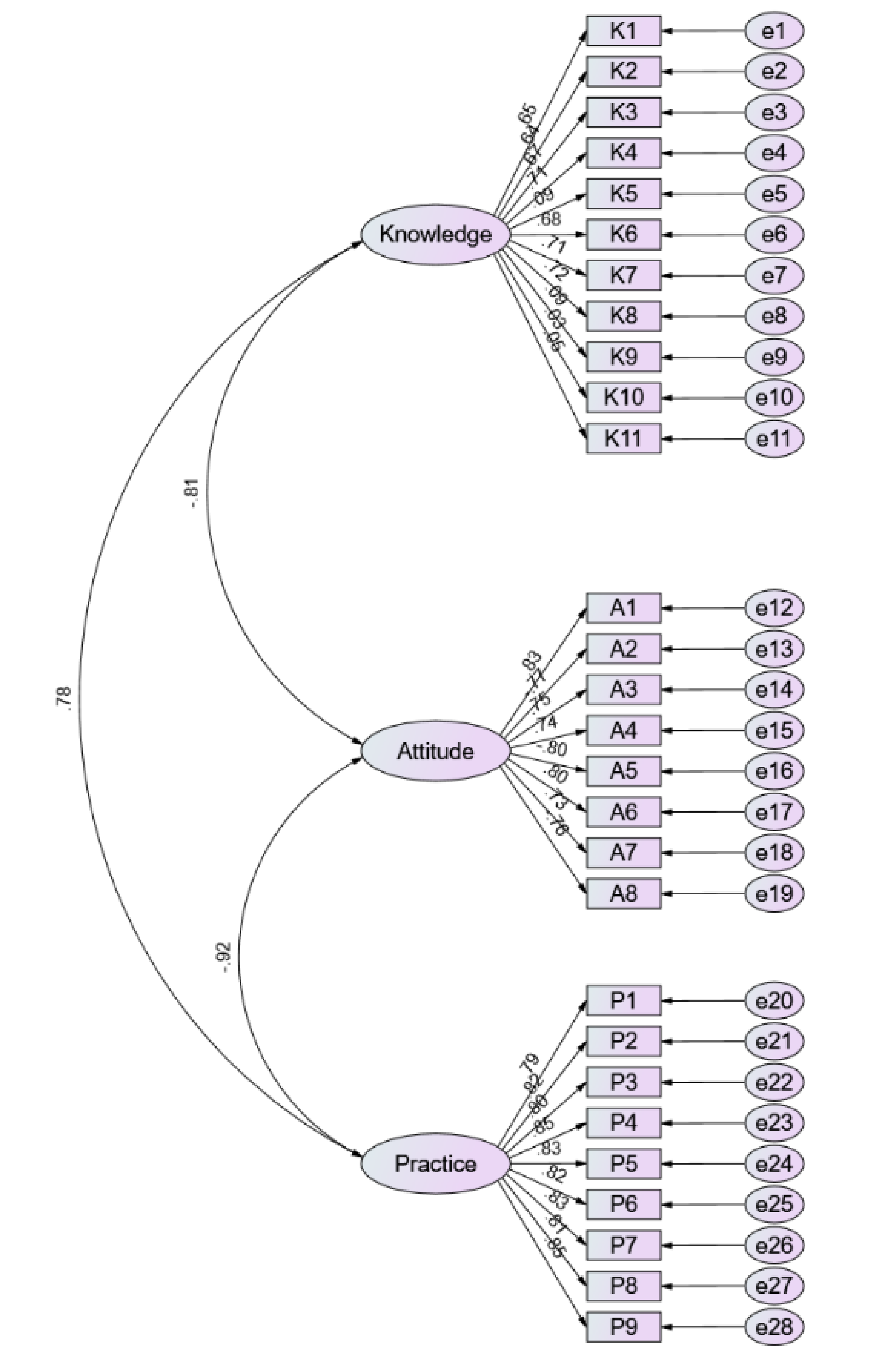

Supplement: Supplementary file 2 [file Image_1.tif]
